# Vertigo from Tobacco

**Published:** 1870-07

**Authors:** 


VERTIGO FROM TOBACCO.—Dr. E. Decaisne in the *Gazette des Hopitaux*, adds another count to the bill of indictment against tobacco. He narrates the case of a gentleman of sixty, long accustomed to the use of tobacco, who was suddenly seized with nervous prostration and violent vertigo. His face was pale, hands and head hot, acid eructations, intermittent pulse, and a sense of heat at the epigastrium. The doctor found that the previous evening he had smoked to excess, and had had a troubled night with bad dreams. A dose of magnesia, a *tisane* of gentian, and rest relieved him, but a month or two after, five segars in an evening brought back all the symptoms. Cured of them again, the patient reduced his allowance to two segars a day, and has since enjoyed good health.

